# Study of Interactions between Saponin Biosurfactant and Model Biological Membranes: Phospholipid Monolayers and Liposomes

**DOI:** 10.3390/molecules28041965

**Published:** 2023-02-18

**Authors:** Monika Rojewska, Wojciech Smułek, Adam Grzywaczyk, Ewa Kaczorek, Krystyna Prochaska

**Affiliations:** Institute of Chemical Technology and Engineering, Poznań University of Technology, Berdychowo 4, 60-965 Poznań, Poland

**Keywords:** adsorption, BAM, DLS, extracts of saponins, FTIR technique, *Glycyrhiza glabra*, impurities, Langmuir monolayer, liposomes, π–A isotherm, relaxation of monolayer

## Abstract

The aim of this study was to determine the effect of saponins-rich plant extract on two model biological membranes: phospholipid monolayers and liposomes. The Langmuir monolayer technique was used to study the interactions of model phospholipid membranes with saponins. The π–A isotherms were determined for DPPE (1,2-dipalmitoyl-sn-glycero-3-phosphoethanolamine) monolayer with the addition of various concentrations of licorice saponins extracts and subjected to qualitative as well as quantitative analysis. Additionally, relaxation studies of the obtained monolayers were carried out and morphological changes were examined using Brewster angle microscopy. Moreover, changes in the structure of phospholipid vesicles treated with solutions of saponins-rich plant extracts were assessed using the FTIR technique. The size and zeta potential of the liposomes were estimated based on DLS methods. The obtained results indicated that the saponins interact with the phospholipid membrane formed by DPPE molecules and that the stability of the mixed DPPE/saponins monolayer strongly depends on the presence of impurities in saponins. Furthermore, it was found that the plant extract rich in saponins biosurfactant interacts mainly with the hydrophilic part of liposomes.

## 1. Introduction

Biosurfactants from the group of saponins have gained significant interest during recent years due to their various biological, therapeutic, and pharmaceutical effects [1,2,3]. Saponins are natural, surface-active glycosides which include a single or several hydrophilic glycoside molecules linked to a lipophilic triterpene molecule. Medicinal plants are the main source used for the preparation and extraction of various modern drugs and pharmaceutical agents, including saponins [4]. Among such plants, licorice is one of the oldest and most widely used herbs, containing more than 20 triterpenoids and 300 flavonoids [5]. Many studies have shown that the active compounds isolated from licorice exhibit anti-cancer, anti-viral, anti-inflammatory, and immunoregulatory effects, as well as several other actions which contribute to the regeneration and protection of the nervous, respiratory, digestive, endocrine, and cardiovascular systems [6]. The notable antibacterial properties of licorice have been emphasized in particular because many studies have reported that aqueous [7], ethanol, and supercritical fluid licorice extracts efficiently inhibit the activity of Gram-positive and -negative bacteria, such as *Staphylococcus aureus* [8], *Escherichia coli*, *Pseudomonas aeruginosa*, *Candida albicans* [9], and *Bacillus subtilis* [10].

Due to such valuable biological properties, the interest in saponins as a bionatural material is growing. Their application in antibacterial therapy is considered in order to facilitate the transport of antibiotics through the biological membrane of bacteria [11,12]. For this reason, the first step to ensure a proper design of saponins–antibiotic mixtures is to elucidate the mechanism of interactions between saponins and bacterial membrane components, such as phospholipids. However, understanding how saponins interact with components of the membrane at the molecular level is a challenging task. Therefore, many studies have been conducted to determine the effects of saponins on the biomimetic systems [12,13,14,15]. These experiments allow to analyze the interactions between the components of a model membrane and biologically active substances using various physicochemical methods.

Biomimetic studies are commonly used as screening tests in laboratory practice during the first stages of an experiment focused on biologically active compounds (potential drugs) and constitute an important step in drug design research [16,17].

Liposomes are spherical, closed structures which consist of a lipid bilayer. The unique structure of these vesicles allows for encapsulation of different substances, both hydrophilic and hydrophobic, in order to deliver them to specific tissues or cells. Due to their flexibility, variety of ingredients, ease of functionalization, tunability of the number of layers/sizes, biocompatibility, and biodegradability, liposomes are widely used in medicine and cosmetics as good carriers for biologically active substances. Their efficiency as a carrier of drugs depends on their physicochemical parameters, such as composition, size, polydispersity, their zeta potential, and the capability of drug loading [18,19]. A structural unit of liposomes (a bilayer) can be considered as a set of two monolayers in which the polar parts of molecules are directed outwards. Hence, the Langmuir monolayer at the air–water interface may be applied to investigate the molecular packing and the interactions between molecules in the mixed vesicle systems [20].

The Langmuir monolayer technique is a very sensitive method used to investigate the interactions of bioactive substances with components of biological membranes [12,21,22,23,24]. A lipid monolayer at the air/water interface represents a promising model surface to study interactions with components dissolved in the adjacent water phase, such as saponins. The monolayer film is formed by spreading organic compounds (e.g., lipids, phospholipids, or glycolipids) on the aqueous subphase. The lipid layer is built by molecules which are specifically oriented at the air/water surface due to their amphiphilic character. The monolayer is compressed by Teflon barriers and the change in surface pressure vs. the monomolecular layer area is monitored. The surface pressure is recorded by a Wilhelmy plate connected to a pressure sensor. The monolayer compressed to the surface pressure of 30 mN/m represents a biological membrane under natural conditions [25,26].

The main aim of this study was to evaluate the ability of naturally occurring triterpenoid saponins to interact and cross a phospholipid membrane which consisted of DPPE (1,2-dipalmitoyl-sn-glycero-3-phosphoethanolamine) molecules. The experiments were prepared for two biomimetic systems: for a monolayer (2D structure) using the Langmuir technique and for liposomes (3D structure). The saponins used in our research were obtained by methanol extraction from *Glycyrhiza glabra* roots. Their chemical structure was previously defined by Schmid et al. [27]. During an attempt to associate the molecular structure with interfacial behavior, it should be kept in mind that the extracts can differ in terms of the saponins content and composition. We expected that impurities, such as residual plant substances, may be present and affect the interfacial properties. Therefore, we have studied two fractions of extracts: a crude extract (GgC) and a purified extract (GgP).

## 2. Results

### 2.1. Interactions with the Monolayer Membrane

#### 2.1.1. The Surface Pressure-Area and Surface Potential-Area Isotherms

**Figure 1:**
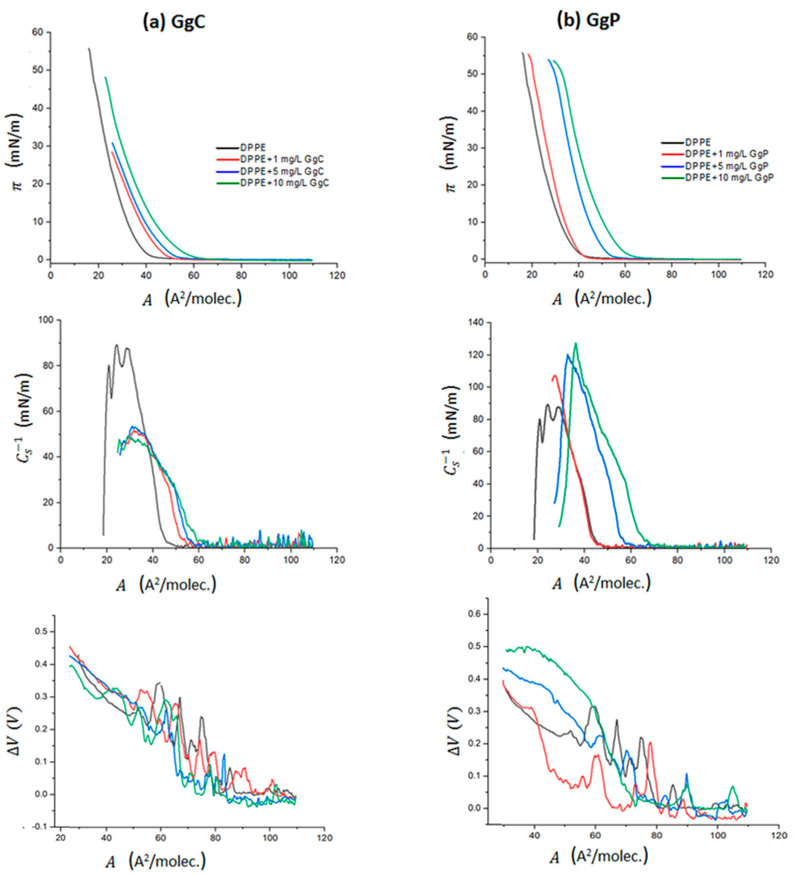
Surface pressure–area per molecule (π–A) isotherms, compression modulus-area per molecule (*C_s_*^−1^–A) graphs and surface potential- area per molecule (ΔV–A) for the analysed systems: DPPE and various concentrations of the crude saponins extract GgC (**a**) and purified extract GgP (**b**).

The Langmuir technique is a unique method used to prepare monomolecular insoluble films of biological substances on aqueous phases, whereby intermolecular interactions as well as their influence on the molecular alignment can be easily determined. In our experiments, the DPPE solution was initially spread on the surface of the subphases. Then, a saponins solution was injected into the subphase one minute prior to the start of compression. Saponins solution (1 g/mL) was added in an appropriate volume in order to achieve the following final subphase concentrations: 1 mg/L, 5 mg/L, and 10 mg/L. For such prepared system, the changes in the surface pressure of the monolayer (π) from the mean molecular area at the air/buffer interface (A) were recorded during film compression. The obtained isotherms are shown in Figure 1. Simultaneously, the surface potential–area per molecule (ΔV–A) isotherms were registered (Figure 1), and the morphology of the mixed monolayer was visualized using a BAM microscope.

Figure 1 illustrates the π–A isotherms obtained for DPPE with two different extracts of saponins, crude (GgC) and purified (GgP). According to the literature [28], the obtained surface pressure–area isotherm of the DPPE monolayer on a D–PBS buffer is almost identical with the π–A isotherm for DPPE on water. For all analyzed systems, π–A isotherms are shifted towards higher values of the surface area A per molecule in the mixed DPPE/saponins monolayer with respect to the isotherm obtained for the DPPE monolayer. The “isotherm lift-off” occurs at different area per molecule values, A_lift-off_, depending on the concentration. The obtained values of the A_lift-off_ parameter, based on the π–A isotherms, are presented in Table 1. The observed shift of the π–A isotherm increases with the increase of saponins concentration in the subphase. For the system with the addition of the highest concentration (10 mg/L) of the GgC extract, we observed an increase in surface pressure at the A_lift-off_ surface of approximately 61 Å^2^/molec, while for the lowest extract concentration A_lift-off_ is equal to approximately 50 Å^2^/molec. Moreover, a more stable monolayer is formed in the presence of a higher concentration (10 mg/L). This film can be compressed to a higher surface pressure, which corresponds to the similar area of molecule at the interface (A_collapse_) for both high and low concentrations. In the case of purified extract, such an effect is not clearly visible, and the created mixed monolayers are characterized by higher A_lift-off_ values than those estimated for the GgC extract. As the concentration of the saponins extract increases, the values of A_lift-off_ also increase from approximately 43 to 65 Å^2^/molec. This effect also indicates a stronger interaction of the molecules in the GgP extract with phospholipids at the interface. Based on the observed effect of a stronger expansion of the film it can be assumed that the content of saponins in the purified extract is definitely higher, as saponins molecules are more strongly incorporated into the lipid film. However, the collapse of the monolayer occurs at lower surface pressure in the presence of a higher concentration of the GgP extract. Detailed information regarding the parameters of the π–A isotherms is given in Table 1.

The estimated values of π_collapse_ for the DPPE monolayer in the presence of the GgP extract oscillate in the range of 53–55 mN/m. Thus, the mixed monolayer formed in the presence of the GgP extract is characterized by similar stability to that of the pure DPPE film. The opposite effect is observed for the GgC extract. The addition of the GgC extract to the subphase caused the collapse of the monolayer which corresponds to the surface pressure in the range 28–48 mN/m, which is notably lower compared to the DPPE film. Therefore, it can be assumed that the presence of impurities strongly affects the stability of the formed mixed monolayers.

The compression modulus values, *C_s_*^−1^ = f(A), were directly calculated based on the π–A isotherm. The modulus is defined as follows [29]:

(1) Cs−1=−A·(dπdA)T

The *C_s_*^−1^ values provide information regarding the physical state of monolayers strictly associated with the ordering and packing of molecules at the air–water interface. The value of *C_s_*^−1^ is assumed as zero for pure air-water interface and increases with the presence of surfactants at the interface. A higher compression modulus value corresponds to a less compressible membrane. According to the Davies and Rideal classification [29], the gas state (G) is in the range of 0–12.5 mN/m, the liquid-expanded (LE) state is characterized by the *C_s_*^−1^ modulus values between 12.5 and 50 mN/m, while the liquid-condensed (LC) state in in the range between 100 and 250 mN/m. The *C_s_*^−1^ values above 250 mN/m refer to a solid state (S) of the monolayer. The maximum values of *C_s_*^−1^ correspond to the most compressed state of the monolayer that is manifested as the “highest peak” point of the *C_s_*^−1^ = f(*A*) function (Figure 1). Based on these maximum *C_s_*^−1^ values (Table 1), it can be established that all mixed monolayers formed in the presence of the GgC extract are mainly in a liquid-expanded (LE) state. On the other hand, it can be observed that the π–A curves of DPPE/GgP systems exhibit a lower slope, which is more characteristic for higher monolayer compressibility than that of the DPPE/GgC systems. In consequence, this effect is reflected by the obtained values of the compressibility modulus, which are in the range of approximately 107 mN/m to 127 mN/m for the DPPE/GgP system. As a result, the compression molecules of DPPE and saponins from the purified extract lead to the formation of films in the LC state. Interestingly, a disparate result was observed for the DPPE film in the presence of both extracts. It can be observed that the degree of impurity strongly affects the surface properties of the phospholipid monolayer. The addition of the GgC extract causes the formation of a more fluidized phospholipid monolayer (characterized by a lower *C_s_*^−1^ value than a pure DPPE monolayer), while the addition of the GcP extract leads to a more condensed structure in reference to the DPPE film.

The surface potential ΔV of a monolayer is defined as the difference in the potential between a clean water surface and a monolayer-covered surface [30]. This quantity depends on both the packing density and the orientation of the molecules [31]. The set of surface potential changes versus area (ΔV–A) for particular systems is presented in Figure 1.

For the analyzed systems, a change in the ΔV value was obtained in different ranges, which proves the reorientation of molecules at the interface during compression of the mixed film DPPE/saponins extract. For the DPPE/GgC system, when the monolayer was compressed to a molecular area A of approximately 65 Å^2^/molec., slight changes in potential were recorded. Only after exceeding this value, a rapid increase in the surface potential value was visible, which corresponds to a rapid increase in surface pressure on the π–A isotherm (Figure 1). This indicates that the molecules are reoriented perpendicular to the surface at the interface and form an increasingly ordered structure at the interface. For the DPPE/GgP system, a more diversified course of ΔV–A curves relative to each other was obtained. The influence of saponins concentration on the course of the surface potential curve is particularly visible. However, in the case of the purified extract GgP, the influence of saponins concentration on the course of the potential curve is much more pronounced.

The greatest increase in the change of the potential value was obtained for the system with the highest saponins concentration, i.e., 10 mg/L. It can be assumed that a higher concentration of surfactant molecules at the interface reduces the available surface area and forces the molecules to strongly reorient to each other during film compression to ensure the best and most favorable packing. This also proves the presence of strong interactions between DPPE molecules and saponins.

#### 2.1.2. Brewster Angle Microscopy Images

**Figure 2:**
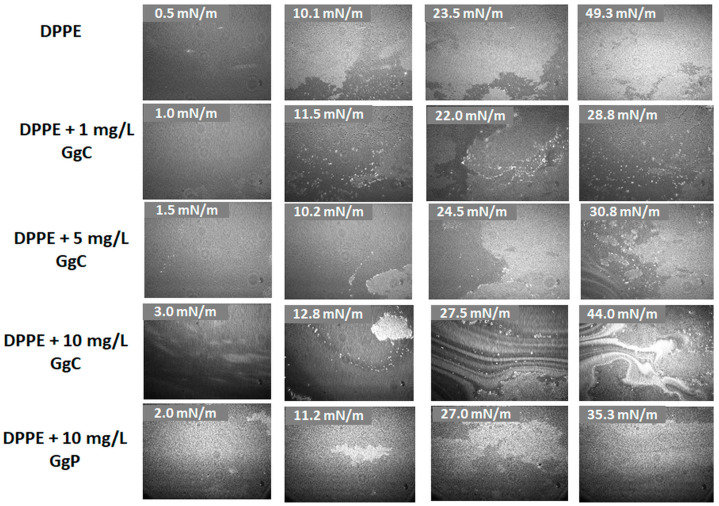
BAM images of DPPE, DPPE/GgC and DPPE/GgP monolayers at selected values of surface pressure, π.

Brewster angle microscopy (BAM) images demonstrate the phase behavior of the Langmuir monolayers mimicking single leaflets of cell membranes. During the compression of the mixed monolayer, a visual analysis of the formed films has been performed. The effect of the GgC concentration on the model membrane structure has been investigated. Figure 2 presents the BAM images of the studied systems at different surface pressure. The obtained BAM images for the DPPE monolayer indicate that the packing of the monolayer increases during the lipid film compression and then a homogeneous film is created. A continuous and densely packed film is reached at approximately 49 mN/m. The observed effect is consistent with the results presented in the literature [28]. It has been shown that the hydrogens in the DPPE ammonium group can form hydrogen bonds with neighboring DPPE headgroups. Therefore, molecules can easily interact with each other to form domains and consequently create a stable DPPE monolayer.

BAM images indicated significant differences in the morphology of the clean DPPE and DPPE film after addition of the extract. Compressing a DPPE film with 1 mg/L of GgC to a surface pressure of approximately 11 mN/m results in the formation of domains that rise up at the lipids films (bright spots). The application of higher concentrations of the crude extract leads to the formation of large aggregates which interact with the lipid homogenous film and cause its disintegration. A two-phase heterogeneous structure was formed for a strongly compressed DPPE/GgC film (approximately 43 mN/m) at the concentration of 10 mg/L (Figure 2) and multi-layer ribbon-like structures were obtained afterwards. However, such a strong differentiation in the surface morphology cannot be observed for the DPPE/GgP system at the same concentration. Therefore, it can be assumed that the formed multilayer structures are mainly the result of the interaction between surface-active impurities present in the GgC extract and phospholipids.

#### 2.1.3. Relaxation/Penetration Studies

**Figure 3:**
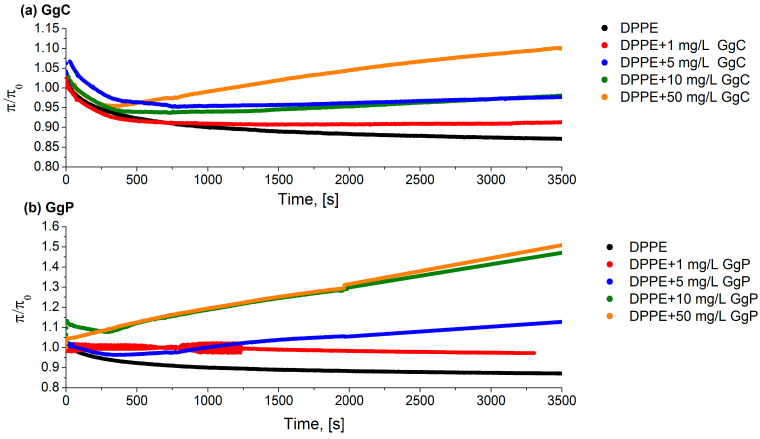
Relative surface pressure-time curves for a various concentration of saponins extracts (**a**) GgC, (**b**) GgP pumped underneath of DPPE monolayer.

The effect of saponins on monolayers was studied by measuring the surface pressure relaxation. The DPPE solution dissolved in chloroform was deposited onto the phosphate buffer and compressed to the initial surface pressure (π = 30 mN/m). Then, the subphase (phosphate buffer) was exchanged for the corresponding extract by a peristaltic pump. Figure 3 shows the relaxation curves for the DPPE monolayers with various concentrations of the extracts. The relaxation experiment consisted of maintaining the molecular area (A) at a constant level and recording the surface pressure (π) as a function of time. When an extract was introduced into the subphase, a change in surface pressure value was observed in the monolayer (π(t)). If the value of (π(t)) is greater than the π_0_ (π(t)/π_0_ > 1), an increase in the area per molecule caused by the embedded saponins molecule in the phospholipid monolayer is indicated. Otherwise, if π(t)/π_0_ < 1, there is a surface pressure loss in the monolayer. Hence, it can be assumed that the phospholipid molecules desorb from the monolayer and dissolve in the subphase. The π/π_0_ parameter was therefore a measure of the monolayer stability. The obtained results clearly show that the process of incorporation of saponins molecules into the model membrane strongly depends on the concentration of the biosurfactant solution. Generally, it was observed that the increase of the saponins concentration in the subphase results in a more rapid incorporation of saponins into the monolayer. In case of both analyzed extracts, the relative value of the surface pressure was above 1, which means that saponins molecules slowly diffuse from the subphase to the phase boundary and are incorporated into the structure of the lipid film. A stronger effect of the interaction of the DPPE monolayer with the extract particles was observed for the GgP solution compared to GgC. The differences are clear in the case of a comparison of extract solutions at 50 mg/L. After 3500 s, the surface pressure of mixed monolayer increased by approximately 10% in the presence GgC solution, while in the case of the GgP system the increase was equal to approximately 50% (see Figure 3).

On the basis of the obtained relaxation curves (Figure 3), it was found that the diffusion rate of molecules to the interface increased with the increase of the concentration of saponins in the system. The diffusivity of the system can be determined on the basis of the obtained slope of the relaxation curve. The greater slope, the stronger the diffusion of molecules from the subphase to the interface. On the other hand, for the GgC systems, it can also be observed that the lack of saponins’ incorporation may result from the insufficient potential of the saponins molecules to expand the structure of the packed DPPE film. The DPPE monolayer at a surface pressure of 30 mN/m is characterized by *C_s_*^−1^ value of approximately 90 mN/m, which corresponds to a film in the condensed liquid phase (LC). The formation of the DPPE film in the condensed liquid phase means an ordered film was formed, in the case of which strong hydrophobic interactions occur between the hydrocarbon chains in the phospholipid molecules and contribute to a tightly packed monolayer. The formation of such a film may constitute a physical barrier that a small amount of saponins molecules are unable to overcome. Thus, they incorporate into the structure of the membrane, which leads to its emulsification. As presented in Figure 3, only a sufficiently high concentration of saponins can enhance the expansion of the compact structure and lead to its disturbance, thus facilitating the transport of other substances, such as drugs. Based on the obtained relaxation curves, it was found that the value of the surface pressure for the DPPE monolayer decreases over time, with a decline in the value of π/π_0_ observed at the beginning of the relaxation process. Thus, the formed monomolecular DPPE film is not stable in the initial phase of the measurement, and the decreasing surface pressure values indicate the loss of adsorbed DPPE molecules from the buffer/air interface to the subphase. The addition of a low concentration of saponins extract to the system results in the formation of a mixed monolayer characterized by greater stability.

Nevertheless, it should be emphasized that the increase of the surfactant concentration in the subphase favors stronger penetration of saponins into the lipid structure of the membrane. Therefore, their excessive concentration may lead to changes in the turgor of cells. This applies not only to the membranes of bacteria, but also the membranes of other organelles in the body. Nowotarska et al. [32] also showed the effect of saponins (i.e., digitonin, tribulosin, dioscin, and escin) on the surface properties of the lipid monolayer of DMPC (dipalmitoyl phosphatidylcholine) enriched with cholesterol. The authors found that saponins strongly interact with the phospholipid film and the additional presence of cholesterol in the DMPC monolayer causes the formation of pores in the film and promotes the adsorption of saponins molecules to the lipid monolayer.

### 2.2. Interaction with a Spherical Model Membrane

**Figure 4:**
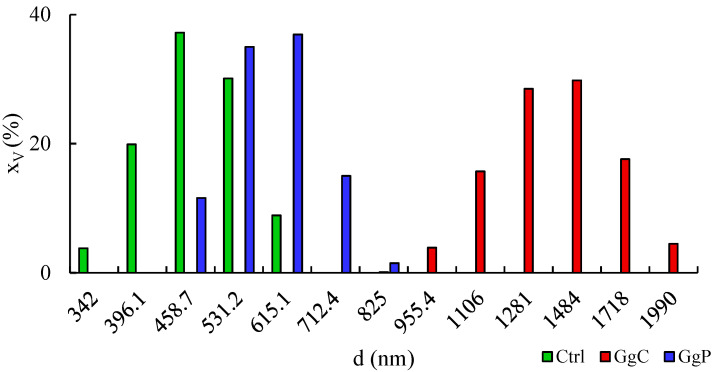
Particle size distribution of liposomes untreated and treated with extracts (x_v_—volume fraction in %, d—hydrodynamic diameter); Ctrl—control sample of DPPE liposomes, GgC—liposomes with *G. glabra* crude extract, GgP—liposomes with *G. glabra* purified extract.

In the next stage of the research, in order to approximate the model more closely to the real system, i.e., the living cell, tests were carried out with spherical systems, i.e., with liposomes. The results indicating the effect of saponins on surface properties of liposomes are presented in Figure 4. The hydrodynamic diameter of untreated liposomes is in the range of 342–615.1 nm with the largest share for 458.7 nm with x_v_ = 37.2%. The addition of 10 mg/mL of GgC induced a shift towards larger particles, from 955.4 nm to 1990 nm. Hydrodynamic diameters, which are equal to 1484 nm with x_v_ = 29.8% and 1281 nm with x_v_ = 28.5%, contribute to the total share to the greatest extent. Moreover, the increase of zeta potential of liposomes from −47 to almost −51.5 mV can be noticed (Table 2), with no noticeable effect on PDI. On the other hand, the purified extract did not cause a significant diameter shift toward larger particles when compared to liposomes exposed to GgC, but the stabilization of obtained suspension can be noticed, as the PDI declined to 0.537. On the other hand, the zeta potential increased to −42.74 mV.

The zeta potential of a model biomembrane in the spherical form is mostly affected by the headgroups of phospholipids, the charges of which are responsible for the distribution of counter ions [2]. The value of zeta potential obtained for DPPE is typical for liposomes with an ethanolamine headgroup. As observed by Taetz et al., DOPE modified with hyaluronic acid and unmodified was in the range from +50 to +60 mV for pH 6.5 [3]. However, there are suggestions that the charge (positive or negative) of zeta potential may simply not be predicted based on the composition of headgroups i.e., it depends on ionic media and phase state [2,4]. Thus, the change in zeta potential value may indicate the disorders in charge distribution on the surface of liposomes. The presence of particles may disrupt the surface charge distribution as saponins are non-ionic surfactants.

Considering the size distribution, and polydispersity index results, the higher hydrodynamic diameter of particles treated with GgC and GgP may be noticed. As Rahnfeld et al. stated, the electrostatic interaction and hydration repulsive forces prevent the aggregation of negatively charged liposomes [5]. Hence, the size of particles may not be correlated with aggregation of liposomes but rather with a tear of liposome structures and joining into bigger vesicles with saponins during their incorporation. The effect of extract purification on the recorded results should also be noted. The shift in particle size toward larger values for the sample with GgC may be due to the greater influence of polymeric compounds (e.g., polysaccharides) on aggregate formation, also with DPPE. Similarly, in the case of zeta potential, it can be seen that the control sample and the sample with GgP exhibit more similar values than the one with liposomes from GgC.

This shows that not only the presence of saponins, but also the proportion of macromolecular compounds are important factors which affect the surface-active properties of natural surfactants.

**Figure 5:**
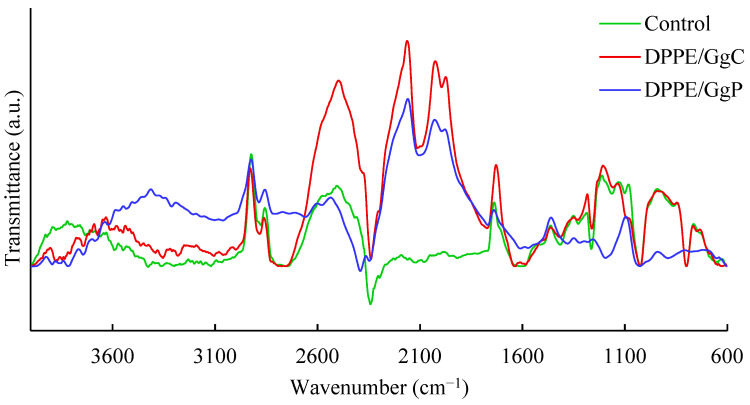
FTIR spectra of DPPE/GgC and DPPE/GgP.

Further important indications were provided by the analysis of infrared spectra of liposome samples, which are presented in Figure 5. Among the outstanding signals, one should point out those at approximately 1730 cm^−1^ originating from the stretching vibrations of the carbonyl group. Significant differences between the spectra can be seen at approximately 1240 cm^−1^ and 1090 cm^−1^, which most likely originate from the P=O bond vibrations of the phosphate group of phospholipids. Changes in these regions may indicate that the compounds present in the GgC and GgP extracts interact mainly with the hydrophilic parts of the phospholipid molecules. Since there are no significant changes in signals from the hydrocarbon chain, it can be assumed that the compounds present in the extracts, including saponins, do not penetrate deeper into the liposome membrane.

## 3. Discussion

Model biological membranes prepared using the Langmuir technique are used to mimic real membrane conditions and represent a useful tool for characterizing membrane component interactions with active substances present in saponins extract at the molecular level. Through the use of a model membrane, the effects of licorice concentrations and its impurities on the surface properties of a lipid DPPE film were investigated. For all analyzed systems, it was established that interactions between saponins and phospholipids occur. This is evidenced by the incorporation of molecules into the structure of the film, which is confirmed by the obtained test results, e.g., shift of the π–A isotherm towards higher surface values as well as an increase in surface pressure during the DPPE relaxation process. Undoubtedly, it has been shown that the surface properties are influenced by the concentration of saponins and the purity of the applied extract. Stronger interaction effects were observed for the purified extract. Moreover, purified extracts GgP introduced into the DPPE monolayer cause the formation of mixed films characterized by greater condensation and packing, as evidenced by the estimated values of maximum compressibility. The obtained results also showed significant interference of saponins in the morphology of the model lipid membrane.

In addition, differences between purified and crude extracts were also found in terms of their interaction with liposomal systems. Changes in particle size distribution and zeta potential show that GgC may contain macromolecular compounds that form relatively large agglomerates. In addition, changes in the infrared spectra may suggest that the interaction of extract components with phospholipids mainly involves their hydrophilic parts, i.e., phosphate groups.

In summary, it can be concluded that the GgP extract exhibited better surface activities in reference to the two-dimensional lipid monolayer. The exclusion of other surface-active compounds present in the extract by filtration promoted the increase of interactions of saponins with lipid molecules. Moreover, filtration of the extract reduces the amount of other surface-active compounds which could be competitive with saponins during adsorption at the interface. The stronger surface-active properties of GgP compared to GgC directly indicate the importance of the extract purification process. The exclusion of large-molecular compounds above 3kDa and small-molecular compounds below 0.5 kDa clearly improved the homogeneity of the plant surfactant composition. Moreover, it can be concluded that the percentage of saponins, as the key group of compounds responsible for the surface properties of the purified extract fraction, increased significantly in GgC.

## 4. Materials and Methods

*Chemicals*

Extract from *Glycyrhizza glabra* roots from FLOS (Mokrsko, Poland) was obtained by methanol extraction in a Soxhlet apparatus. Then, the extract was subjected to two nanofiltration processes, first using a 3-kDa (Merckmilipore, Darmstadt, Germany) membrane, then, the second, with the use of a 0.5-kDa membrane (TriSep, Sterlitech, Auburn, WA, USA). The process was conducted using Amicon Stirred Cell with a total volume of 200 mL (Merkmilipore). As a result, two fractions of extracts were used during the research, crude extract marked as GgC and purified extract (with molecules size between 3 kDa and 0.5 kDa), GgP.

In the presented studies, 1,2-dipalmitoyl-*sn*-glycero-3-phosphoethanolamine (DPPE, 99%; from Avanti Polar Lipids (Alabaster, AL, USA)) was used as the film and liposome forming substance. Chloroform of high-purity Uvasol (Merck, Warszawa, Poland) was used to prepare the Langmuir monolayer. Dulbecco′s Phosphate Buffered Saline (Sigma Aldrich, Poznań wielkopolskie, Poland) was applied as a solvent to prepare saponins solution or as a subphase.

Deuterium oxide was purchased from Merck KGaA (Darmstadt, Germany).

### 4.1. Research Regarding Monolayer Membranes

*π–A isotherms*

The subphase was placed in a Teflon trough (KSV Nima, Helsinki, Finland) with a surface area of 238 cm^2^. During the measurements, the temperature was kept constant at 25.0 ± 0.1 °C with a Julabo circulator. Before each measurement, the subphase was cleaned to the surface pressure below π = 0.35 mN/m reached in maximum compression.

First, the DPPC solution (with c = 1 mg/mL) was spread on the buffer surface with a Hamilton microsyringe (25 µL), and chloroform was allowed to evaporate for 15 min. One minute before the compression of the monolayer was started, the appropriate concentration of saponins solution was injected into the well and stirred. The monolayer was compressed by symmetrical movement of the barriers with a velocity of 10 mm/min.

The surface pressure π (mN/m) was measured as the function of the area per DPPE molecule A (Å^2^/molec.).

*Surface potential measurements*

The surface potential (∆V) was measured simultaneously with surface pressure using surface potential sensor. The non-destructive, non-contact vibrating capacitor method was applied (SPOT; KSV Nima). The instrument worked with two electrodes: the first immersed in the subphase and the vibrating electrode located just above the water surface. The surface potential was measured with the sensitivity of ±1 mV.

*Brewster angle microscopy*

Brewster angle microscopy (MicroBAM; KSV Nima) was used to visualize the monolayer morphology. The images were captured during the monolayer compression. A black glass plate was placed under the subphase to absorb the refracted beam. The resolution of the image was equal to approximately 6 microns pixel^−1^.

*Relaxation measurements*

The relaxation of the DPPE film was observed when additional molecules were introduced into the monolayer using a peristaltic pump (MINIPULS 3, Gilson, Middleton, WI, USA). After formation of the DPPE monolayer, the subphase (DPBS buffer) was replaced with a new solution which contained various concentrations of saponins. A detailed description of the measurement has been provided in our previous publication [12]. The relaxation experiments were performed for the pure DPPE monolayer and mixed systems: DPPE/saponins. The DPPE film was initially compressed to a desired surface pressure of 30 mN/m and after that the solution of saponins was pumped underneath the film to the subphase. The observed changes in surface pressure were recorded over time. The obtained results were presented as π/π_0_, which is the ratio of the actual surface pressure in time *t* to the surface pressure at the moment of injection at a constant molecular area between barriers. The temperature of the experiments (25 °C) was kept constant and controlled during measurements by a Julabo F-12 circulator (Cole-Parmer, Wertheim-Mondfel, Germany).

### 4.2. Research Regarding Spherical Membranes

*Liposomes preparation*

Liposomes were prepared as described by Costa et al. [33]. Briefly, DPPE was dissolved in chloroform, followed by evaporation of the organic solvent in a nitrogen stream to obtain a thin phospholipid film. Then, 5 mg/mL of the extract in H_2_O PBS buffer was used for hydronation of the obtained film, to achieve a final concentration of liposomes equal to 2.5%. Then, the solution was subjected to 10 freeze-thaw cycles as a form of homogenization of the systems. For FTIR measurements, H_2_O was replaced by D_2_O.

*FTIR*

Fourier-transform infrared spectroscopy–attenuated total reflectance (FTIR–ATR) was used to analyze the possible wavelength shift as a result of interaction between saponins and phospholipids. In this method, infrared radiation with a vibration frequency close to that of the particles’ bonds is used. Because of that, radiation which penetrates the tested sample is absorbed selectively and increases the vibration amplitude of the functional groups. This allows the characteristic absorption spectrum to be obtained. Measurements was conducted for liposomes suspended in D_2_O/PBS using Vertex 70 (Bruker, Billerica, MA, USA).

*Zeta & Size*

The hydrodynamic diameter of the particles was measured by using the dynamic light scattering method. Zeta potential was calculated using the Smoluchowski equation based on electrophoretic light scattering results. Both analyses were conducted Zetasizer Nano ZS (Malvern Panalytical, Malvern, UK). Measurements were performed at a stable pH equal to 7.4.

## 5. Conclusions

Studies carried out using the Langmuir technique as well as the experiments with liposomes allow to conclude that the tested plant extracts rich in saponins interact with phospholipids present in model biological membranes. However, the strength of these interactions is determined by both the concentration of saponins and the purity of the extract used. The addition of the purified extract to the phospholipid forms a mixed monolayer. It has been proven that the presence of saponins affects the structure and morphology of the model phospholipid membrane, both in the case of flat membrane and spherical liposomes. The results may suggest that the effects of natural surfactants on membranes are mainly caused by their interaction with the hydrophilic part of liposomes.

## Figures and Tables

**Table 1:** Characteristic parameters of π–A isotherms: A_lift-off_—lift-off area of surface pressure. A_collapse_—area corresponding to the monolayer collapse, π_collapse_—collapse pressure [mN/m], max. C_s_^−1^—maximum value of the compression modulus [mN/m] (refers to A_max_ or π_max_).

	A_lift-off_ (Å^2^/molec.)	A_collapse_ (Å^2^/molec.)	π_collapse_ (mN/m)	A_max_ (Å^2^/molec.)	π_max_ (mN/m)	C_s_^−1^_max_ (mN/m)
DPPE	42.4	17.1	56.8	24.2	37.5	89.3
DPPE/1 mg/L GgC	50.2	24.2	28.1	31.6	18.9	51.2
DPPE/5 mg/L GgC	56.8	24.9	30.7	30.8	22.5	53.4
DPPE/10 mg/L GgC	61.2	21.8	48.3	29.2	24.3	49.3
DPPE/1 mg/L GgP	43.5	18.8	55.3	27.6	28.6	107.3
DPPE/5mg/L GgP	57.2	28.6	53.9	32.8	40.7	120.3
DPPE/10 mg/L GgP	64.9	29.8	53.3	36.3	40.6	127.3

**Table 2:** Zeta potential and Polydispersity Index (PDI) of samples, Ctrl—control sample of DPPE liposomes, GgC—liposomes with *G. glabra* crude extract, GgP—liposomes with *G. glabra* purified extract.

Sample	Zeta Potential ζ (mV)	PDI (-)
Control	−46.94 ± 3.06 ^a^	0.800 ± 0.141 ^a’^
GgC	−51.40 ± 0.89 ^b^	0.797 ± 0.143 ^a’^
GgP	−42.74 ± 1.77 ^c^	0.537 ± 0.153 ^a’^

Values marked with the same letter do not differ significantly (*p* > 0.05).

## Data Availability

All data are available from the authors on request.

## Sample Availability

Samples of the saponins-rich plant extracts are available from the authors.
